# A Case of Glaucoma

**Published:** 1893-01

**Authors:** A. G. Allan

**Affiliations:** Philadelphia, Pa.


### A CASE OF GLAUCOMA.

A. G. Allan, M. D., Philadelphia, Pa.

I have presented this case in the Bureau of Surgery instead of Clinical Medicine, because the allopathic treatment of glaucoma is purely surgical. Their method is to perform iridectomy or sclerotomy. In their hands medicines are of no avail; the knife alone is their only resort. But it will be seen by the results in this case that medicines can control this disease.

I have always looked upon much of the pain and disastrous consequences of glaucoma as due more to pressure which seems almost mechanical than to a purely dynamic disturbance, and many of the unsatisfactory results of medicinal treatment I felt constrained to attribute to that cause.

But the result in this case shows that the similar remedy is capable not only of removing the violent subjective symptoms that give the patient such severe suffering, but that it can reduce the tension and accomplish what not even the knife can—a cure of the disease.

November 5th, 1891.—Mrs. W., aged sixty-one, presented herself for treatment suffering with an attack of acute glaucoma of the right eye. She was taken suddenly seven weeks previous to this date with violent pain, photophobia, redness and watering of the eye. She had immediately consulted her family physician, in the small country town where she resided, and he, though a homœopathist, had resorted to palliation, as well as to internal medication. Finally, recognizing the inability of his treatment to cure the case, he advised her to go to Philadelphia and have iridectomy performed as a last resort. Her sufferings, except when thoroughly under the influence of Morphine, were, up to date, what she called almost impossible to bear.

The following is the record of the case from day to day: Right eye, lens hazy, with greenish pupil; pupil dilated; iris greenish; anterior chamber very shallow. Tension—eyeball as hard as stone. Eyeball very red, sharp, shooting pain coming and going suddenly; sharp, shooting pain extending into forehead, attended with a sudden gush of tears; aching in eyeball, worse on moving the lids; lachrymation scalding and hot to the eyeball; upper lid swollen, œdematous, of a deep red color, and opens with difficulty; photophobia; aggravation of symptoms in a bright light; frequent sneezing; during the attacks of sneezing the lids of right eye feel as if they everted with terrible pain in the eyeball and forehead; no vision; can distinguish light from darkness. Received Bell.$^{cm}$, seven doses, half an hour apart. Took first dose at five P. M.

November 6th, 1891.—She seemed to be worse during the evening but fell asleep at eleven P. M., and slept all night and awoke *free from pain*. Less redness; not so much soreness on winking; no sneezing; no lachrymation. Eyeball not nearly so red.

November 7th, 1891.—Tension not so hard. No pain, lachrymation, or photophobia. No soreness on winking; no scalding by tears. Eyeball scarcely red at all. Had some slight smarting in the eyeball early in the morning.

November 9th, 1891.—Continued improvement. Tension; can feel slight elasticity in the eyeball. Eyeball, scarcely any redness discernible. Lid not nearly so swollen and can open nearly as wide as the other eye; color of the lid not so dark.

November 11th, 1891.—Tension the same as on the ninth instant. Eyeball redder, had some pain in it during the night, toward morning probably about three A. M. Some watering. Gave Bell.$^{cm}$, one dose.

November 12th, 1891.—Improved. No redness about the cornea. No pain. Lid the same color as that of the other eye; can open eye same as the other; no lachrymation or photophobia.

November 14th, 1891.—Improved eyeball looks clearer white. Tension diminished; no congestion about cornea.

November 16th, 1891.—Eye same as last time; tension same; iris nearly the same color as that of the left eye; pupil smaller, can see slight reaction under the stimulus of light; pupil now has the same black appearance as that of the left eye.

November 17th, 1891.—Eye looks redder and watery again. Had some pain at three A. M. with heavy feeling. Stormy weather.

November 18th, 1891.—Eye still red and watery, otherwise no change since last here. Gave Bell.$^{cm}$, one dose.

November 20th, 1891.—Eyeball not so red; does not water. Lid does not feel heavy.

November 25th, 1891.—Eyeball much more congested. The lid looks heavy; some photophobia and watering, and some pain at long intervals in eyeball and forehead. Besides she complains of the following new symptoms: sweating over the body at night, feels too warm in bed, pushes off the covers to get cool, but soon has to cover up again lest she chills; sensitive to draughts of air. When she sweats notices eye waters more and feels worse. Feet get cold, does not notice it herself, but they feel cold to others when touched. Feet cold at night; when they are cold upper part of the body feels hot. Feet get cold, also cold legs as far as to the knees. Pain in the tibia worse when it rains and at night and when where it is *very* warm. Soles of feet burn, wants to put them out of bed to cool them. Pains worse from motion. Eye pains and symptoms are all worse when where it is warm, as about the kitchen fire—she cannot do any cooking on this account. Eye pains and symptoms also worse during night and in bed. Since Belladonna has always been followed by good results, gave another dose of Bell $^{cm}$, although the symptoms seemed to point very strongly to Mercury.

November 28th, 1891.—No improvement. Apparently in *statu quo*. Gave another dose of Bell.$^{cm}$.

November 30th, 1891.—Eye looks worse. Tension has not increased but remains the same. Belladonna had evidently done all the good it could, so gave Merc-viv.$^{cm}$, one dose.

December 1st, 1891.—Eye feels better but appears about the same as yesterday.

December 2d, 1891.—Eye appears *very* much better, not nearly so much pericorneal congestion. No pain or photophobia. Can sleep better, does not perspire by night.

December 3d, 1891.—Very much improved. The pericorneal injection is very slight. Tension much less than at any time; can hardly tell whether the tension is increased or not. Pupil is smaller than at any time and contracts and expands perceptibly. Color of iris is more normal than at any time previously.

December 5th, 1891.—Eyeball redder than when last here. Some lachrymation. Had some shooting pain in eye last night (stormy all day yesterday). Gave Merc-viv.$^{cm}$.

December 8th, 1891.—Eye looks much better than at any time since she has been under treatment. Tension still diminished. Pupil smaller. Color of iris the same as that of the other eye. Iris responds some to light.

December 10th, 1891.—Eye does not appear as well as it did the last time she was here. Somewhat watery looking.

December 11th, 1891.—Eye appears better.

December 12th, 1891.—Eye appears the same. She had a dull pain over the eye and in the forehead on awakening, so gave Merc-viv.$^{cm}$ (F.). Since the 8th instant the eye has, with the exception of a partial dilatation of the pupil, appeared normal. At this time the epidemic of influenza was at its height; some members of her family were sick, and she was very anxious to get my consent to go home to take care of them. As she never returned and I was unable to get any word from her, I inferred that she had returned home on her own responsibility.

If we look carefully at the notes of this case, we shall see that the remedies given actually controlled the disease, so we have a demonstration of the ability of the potentized remedy to cure glaucoma.

But there is another point to be observed. It is the custom to give the patient drops to put into the eye made of Eserine, an alkaloid extracted from physostigma, to reduce the tension. I wish to say that if you want to have the highest potencies do any good in this disease you must not use Eserine locally. It takes very little to disturb the action of a potency. I have positive evidence of their action being suspended by even so trivial a thing as a hearty meal, being a long time in a crowded room, and fatigue from a long walk. The tension, we see by our case, can be reduced by the natural reaction from the remedy much better than by Eserine. At the last few visits of our patient, after the administration of the second dose of Merc. especially, it was impossible to discover any increase in the tension whatever. If the remedy will accomplish this, then such local measures as Eserine should be discarded as superfluous and obsolete. For all topical applications make an impression upon the vital-force, rendering the action of the internal medicine uncertain. In such a disease as glaucoma we cannot afford to trust to an uncertainty. We know our potencies and what they can do when not interfered with, and we know they can be trusted, which is more than we can say of any method borrowed from our friends the allopaths.

### Discussion.

Dr. Wesselhœft—What was the condition of the sight after the administration of the medicine?

Dr. A. G. Allan—When the woman came to me there was very little sight indeed. She could tell light from darkness, but that was all. Afterward, she was able to tell when a hand passed between her face and the window. It was a case of very acute glaucoma. The nerve was injured to such an extent that repair was impossible. The whole interior of the eye was hazy, and the pupil greenish. The iris was pressed forward. After treatment, the pupil contracted to very nearly the same size as that of the other eye, the anterior chamber became deeper, and the iris remained bulged forward but very little.

